# The risk of consequent nephropathy following initial weight loss in diabetic patients treated with sodium glucose cotransporter 2 inhibitors

**DOI:** 10.1186/s12933-021-01361-z

**Published:** 2021-08-16

**Authors:** Yi-Hsin Chan, Shao-Wei Chen, Tze-Fan Chao, Yi-Wei Kao, Chien-Ying Huang, Pao-Hsien Chu

**Affiliations:** 1 The Cardiovascular Department, Chang Gung Memorial Hospital, Linkou, Taoyuan, 33305 Taiwan; 2 College of Medicine, Chang Gung University, Taoyuan, 33302 Taiwan; 3 Microscopy Core Laboratory, Chang Gung Memorial Hospital, Linkou, Taoyuan, Taiwan; 4 Division of Thoracic and Cardiovascular Surgery, Department of Surgery, Chang Gung Memorial Hospital, Linkou Medical Center, Chang Gung University, Taoyuan City, Taiwan; 5 Center for Big Data Analytics and Statistics, Chang Gung Memorial Hospital, Taoyuan, Taiwan; 6 Division of Cardiology, Department of Medicine, Taipei Veterans General Hospital, Taipei, Taiwan; 7 Institute of Clinical Medicine, Cardiovascular Research Center, National Yang Ming Chiao Tung University, Taipei, Taiwan; 8 Graduate Institute of Business Administration, College of Management, Fu Jen Catholic University, Taipei, Taiwan

**Keywords:** Type 2 diabetes mellitus, Sodium glucose cotransporter-2 inhibitor, Chronic kidney disease, Estimated glomerular filtration rate, End-stage kidney disease, Obesity

## Abstract

**Background:**

There is a controversy over the association between obesity and the risk of renal events in patients with type 2 diabetes mellitus (T2DM). Furthermore, whether body weight (BW) loss following sodium glucose cotransporter 2 inhibitor (SGLT2i) treatment associated with risk of adverse renal events is unknown.

**Methods:**

We used medical data from a multi-center healthcare provider in Taiwan, enrolling 8992 T2DM patients with a baseline/following-up BW data available after around 12 weeks of SGLT2i treatment, from June 1, 2016 to December 31, 2018. Patients were followed up until the occurrence of composite renal outcome (estimated glomerular filtration rate decline > 40% or end-stage kidney disease) or the end of study period, whichever occurred first.

**Results:**

Participants were divided into six baseline BMI categories: < 18.5 (n = 55); 18.5–22.9 (n = 985); 23.0–24.9 (n = 1389); 25.0–29.9 (n = 3941); 30.0–34.9 (n = 1973); and ≥ 35.0 kg/m^2^ (n = 649). There were 38.9%, 23.5%, 24.7%, 8.4%, 2.7%, and 1.8% of patients experienced no-BW loss, initial BW loss of 0.0–2.4%, 2.5–4.9%, 5.0–7.4%, 7.5–9.9%, and ≥ 10.0%, associated with SGLT2i treatment, respectively. Compared with patients with normal BMI (BMI: 18.5–22.9 kg/m^2^), underweight (BMI: < 18.5 kg/m^2^) was associated with a higher risk of composite renal outcome (adjusted hazard ratio (aHR) [95% confidence intervals (CI)]: 2.17; [1.16–4.04]), whereas pre-obese (BMI: 25.0–29.9 kg/m^2^) associated with the lowest risk of composite renal outcome (0.52; [0.40–0.68]) after multivariate adjustment. Compared with those without BW loss after SGLT2i treatment, BW loss of 0.0–2.4% (0.55; [0.43–0.70]) and 2.5–4.9% (0.78; [0.63–0.98]) were associated with a lower risk, whereas BW loss ≥ 10.0% associated with a higher risk of composite renal outcome (1.61; [1.06–2.46]) after multivariate adjustment.

**Conclusion:**

A modest BW loss of 0–5% associated with SGLT2i treatment was associated with a favorable renal outcome. Caution should be taken for whom are underweight at baseline or have a pronounced BW loss ≥ 10.0% associated with SGLT2i treatment, which was associated with a worse renal outcome.

**Supplementary Information:**

The online version contains supplementary material available at 10.1186/s12933-021-01361-z.

## Background

Type 2 diabetes mellitus (T2DM) is the leading cause of end-stage kidney disease (ESKD) in developed countries [1]. The number of T2DM related ESKD has increased rapidly worldwide [1–3], and ESKD patients comorbid with T2DM have an extremely higher rates of morbidity and mortality when compared with those without T2DM [4, 5]. Sodium-glucose cotransporter 2 inhibitor (SGLT2i) is a new class of anti-diabetic drug functioning through a novel mechanism of inhibiting renal tubular sodium and glucose reabsorption without stimulating insulin release in patients with T2DM [6]. Furthermore, large randomized placebo controlled trials have demonstrated that SGLT2i reduced the risk of adverse renal events consistently when compared with current standard-care of treatment among patients with T2DM, regardless of the presence or absence of chronic kidney disease (CKD) [7–11]. It is noted that SGLT2i augments tubule glomerular feedback through increasing distal tubular sodium and chloride delivery to the macula densa, resulting in an reversal of afferent arteriolar vasodilatation [12]. As a result, use of SGLT2i resulted in an acute decline in intraglomerular pressure and estimated glomerular filtration rate (eGFR) by ~ 3% [12], which is followed by a long-term stabilization of kidney function decline thereafter [7–9, 12].

Although growing evidence have shown an positive association between obesity and risks of adverse kidney events in general population regardless of the presence of T2DM [13–17], few have been specifically focused on population with T2DM [15, 17, 18]. Conversely, some observational studies have shown an inverse association between obesity and CKD or ESRD among population with T2DM [19, 20]. There is a controversy over the association between obesity and the risk of adverse renal events in patients with T2DM. Furthermore, treatment of SGLT2i directly resulted in body weight (BW) loss through glucose excretion via kidney (calorie loss); however, whether BW loss following SGLT2i treatment affects the consequent risk of adverse renal events in patients with T2DM remains unclear. Therefore, the primary aim of the present study was to investigate the association between baseline BMI as well as a different degree of initial BW loss following SGLT2i treatment with the consequent risk of adverse renal event, specifically focused on Asian population with T2DM, in a large real-world setting.

## Methods

### Database

This present study was approved by the Institutional Review Board of the Chang Gung Medical Foundation. The informed consent was waived for this study, because the identification number of each patient is encrypted and de-identified using a consistent encryption procedure. The data was come from the Chang Gung Research Database provided by Chang Gung Memorial Hospital (CGMH). The interpretation and conclusions presented in the present study do not represent the position of CGMH. The CGMH Medical System is currently the largest health care provider in Taiwan, which composed of two medical centers, two regional hospitals, and three district hospitals, with ~ 280,000 admissions per year and a total of 10,050 beds [21]. The advantage of the CGMH medical database is that it contains detailed medical data including diagnoses, medications, interventions, laboratory examinations, and imaging for each patient [21].

### Study design and cohort

**Fig. 1:**
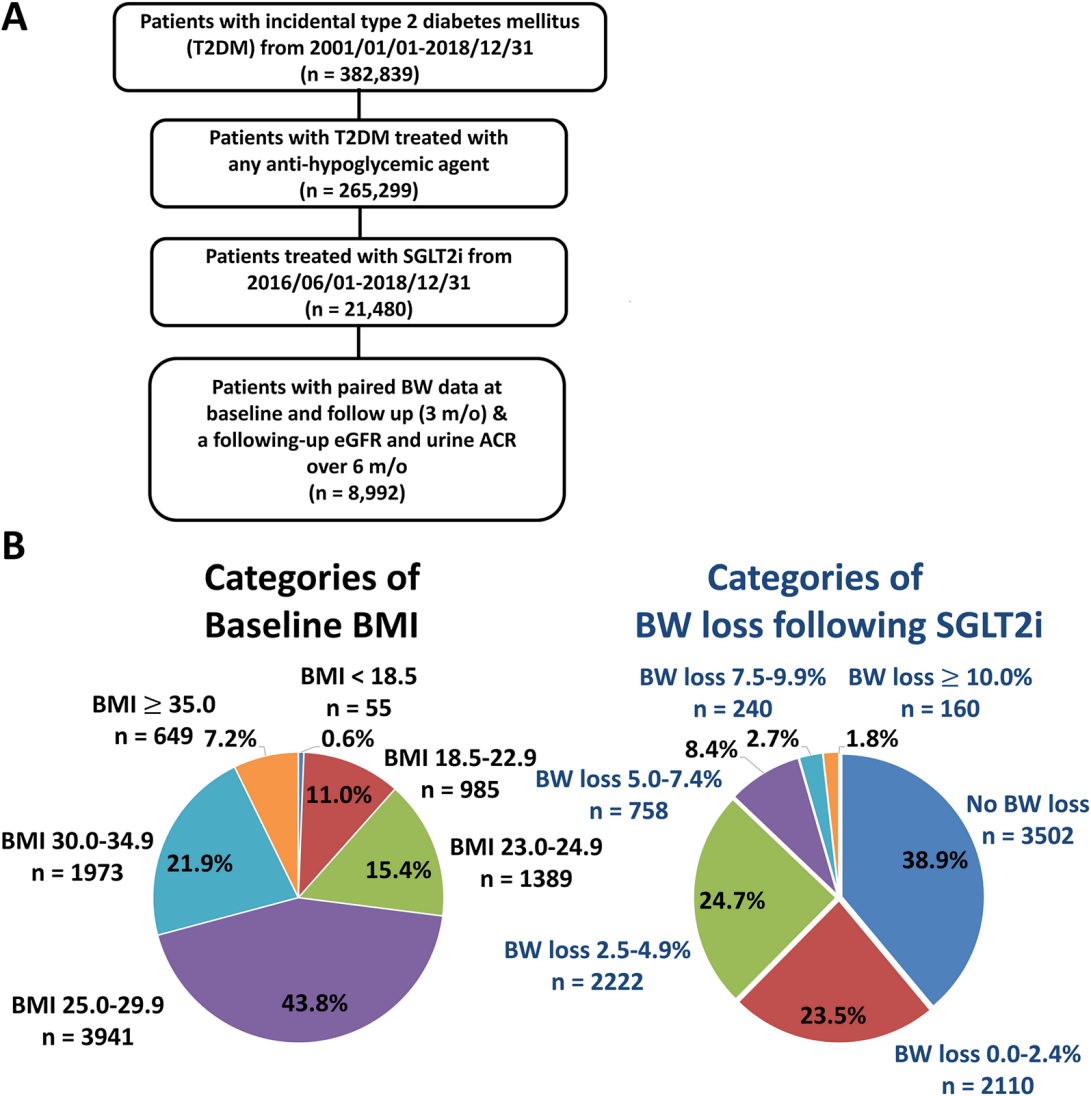
Enrollment of patients with T2DM treated with SGLT2i. A total of 8,992 patients with T2DM treated with SGLT2i from June 1, 2016, to December 31, 2018, were recruited (**A**). We stratified the patients based on their baseline BMI into the following groups: underweight (BMI < 18.5 kg/m^2^), normal (BMI: 18.5–22.9 kg/m^2^), overweight (BMI: 23.0–24.9 kg/m^2^), pre-obese (BMI: 25.0–29.9 kg/m^2^), obese I (BMI: 30.0–34.9 kg/m^2^), and obese II (BMI: ≥ 35.0 kg/m^2^) subgroups. There were 38.9%, 23.5%, 24.7%, 8.4%, 2.7%, and 1.8% of patients experiencing no BW loss, an initial BW loss of 0.0–2.4%, 2.5–4.9%, 5.0–7.4%, 7.5–9.9%, and ≥ 10.0% following around 3 months of SGLT2i treatment (**B**). *ACR* albumin to creatinine ratio, *BMI* body mass index, *BW* body weight, *eGFR* estimated glomerular filtration rate, *SGLT2i* sodium–glucose cotransporter 2 inhibitor, *T2DM* type 2 diabetes mellitus

This was a retrospective and observational study. The study design and flowchart of patient enrollment was shown in Fig. 1A. There were 382,839 patients aged ≥ 20 years in whom new-onset T2DM was diagnosed from January 1, 2001, to December 31, 2018. Those patients who did not use any antidiabetic drugs (n = 117,540) was excluded from the present study. There were 21,480 patients having a first prescription for a SGLT2i (approval date: June 1, 2016) enrolled in the study cohort. We used the BW data nearest to the date of 12 weeks (3 months) after drug index date as the following-up BW data after SGLT2i treatment (mean (SD): 3.04 $\pm$ 1.02 months). Those patients without baseline and following-up BW data after SGLT2i treatment were excluded (n = 9151). We also excluded patients with a minimum follow-up period of eGFR data for < 6 months (n = 8732). Those patients without baseline and following-up urine albumin to creatinine ratio (ACR) were also excluded (n = 10,759). Finally, there were 8992 SGLT2i users with paired BW data and follow-up eGFR and urine ACR enrolled for the final analysis.

We stratified the patients based on their baseline BMI into the following groups: underweight (BMI < 18.5 kg/m^2^; n = 55), normal (BMI: 18.5–22.9 kg/m^2^; n = 985), overweight (BMI: 23.0–24.9 kg/m^2^; n = 1389), pre-obese (BMI: 25.0–29.9 kg/m^2^; n = 3941), obese I (BMI: 30.0–34.9 kg/m^2^; n = 1973), and obese II (BMI: ≥ 35.0 kg/m^2^; n = 649) subgroups, modified from the WHO Asian BMI classifications [22]. Patients were also divided into six groups according to the amount of BW loss following SGLT2i treatment: no BW loss (n = 3502) and BW loss of 0.0–2.4% (n = 2110), 2.5–4.9% (n = 2222), 5.0–7.4% (n = 758), 7.5–9.9% (n = 240), and ≥ 10.0% (n = 160) (Fig. 1B).

### Definition of study outcomes

We calculated the eGFR using the CKD-EPI equation that is as accurate as the MDRD Study equation at GFR less than 60 ml/min/1.73 m^2^ but more accurate at higher GFR [23]. As a primary outcome, the incidence of sustained renal worsening defined as a greater than 40% decline of eGFR from the baseline value or the development of ESKD (eGFR < 15 mL/min/1.73 m^2^) were combined into a composite renal endpoint and compared across study groups [24, 25].

### Covariates

Baseline characteristics referred to any claims record with the aforementioned diagnoses or medication codes prior to the drug index date. A history of any prescription medicine was confined to medications taken at least once within 3 months preceding the index date. Baseline laboratory data listed in Table 1 and Additional file 1: Tables SI and SII were based on the measurements performed within 1 year before the drug index date.

**Table 1:** Clinical characteristics of patients with type 2 diabetes mellitus (T2DM) treated with SGLT2i

	Patient enrollment (n = 8992)
Clinical characteristics	
Diabetes duration (year)	8.6 ± 3.4
Age (year)	58.8 ± 11.3
Female	3864 (43)
Ischemic heart etiology	633 (7)
Hypertension	6032 (67)
Dyslipidemia	7026 (78)
Cerebral vascular accidents	361 (4)
Congestive heart failure	291 (3)
Peripheral artery disease	74 (1)
Gout	924 (10)
Malignancy	734 (8)
Baseline body weight (BW) and BW change	
Baseline body weight (kg)	74.0 ± 15.1
Baseline BMI (kg/m^2^)	27.9 ± 4.7
Body weight change (kg)	− 1.24 ± 2.83
Body weight change (%)	− 1.65 ± 3.84
Baseline laboratory data	
HbA1c (%)	8.9 ± 1.6
eGFR (ml/min/1.73 m^2^)	93.3 ± 22.0
Triglycerides (mg/dL)	180.3 ± 204.7
LDL (mg/dL)	92.5 ± 29.7
HDL (mg/dL)	43.9 ± 11.2
Uric acid (mg/dL)	5.7 ± 1.4
Urine ACR (mg/g)	236.9 ± 787.8
Baseline medications	
Anti-platelet agent	2809 (31)
Statin	5703 (63)
ACEI or ARB	5371 (60)
Use of diuretics	787 (9)
Anti-diabetic agent	
SU	6203 (69)
Metformin	8229 (92)
Glinide	297 (3)
DPP4i	4564 (51)
Glitazone	2340 (26)
Acarbose	1955 (22)
Insulin	1736 (19)
GLP1 agonist	84 (1)

Data are expressed as mean ± standard deviation or number (%)

*ACEI *angiotensin-converting enzyme inhibitor, *ACR *albumin to creatinine ratio, *ARB *angiotensin receptor blocker, *BMI *body mass index, *DPP4i *dipeptidyl peptidase-4 inhibitor, *eGFR *estimated glomerular filtration rate, *GLP1 *glucagon-like peptide 1, *HBA1c *hemoglobin A1c, *HDL* high-density lipoprotein, *LDL* low-density lipoprotein, *SGLT2i *sodium–glucose co-transporter-2 inhibitor, *SU* sulfonylurea

### Statistical analysis

Data are presented as mean ± standard deviation for continuous variables and as proportions for categorical variables. Analysis of variance was used to compare differences in continuous variables, and χ^2^ test was used to compare the differences in nominal variables. Crude incidence rates were computed as the total number of study outcomes during the follow-up time divided by person-years at risk. Multivariate Cox proportional hazards regression was used to compare the risk of events in patients with T2DM across different categories based on baseline body mass index (BMI) or BW loss following SGLT2i treatment. Risk of study outcome was adjusted for age, gender, diabetic duration, baseline comorbidities including history of ischemic heart disease, hypertension, stroke, heart failure, peripheral artery disease, gout, malignancy, and baseline BMI, urine ACR, hemoglobin A1c (HbA1c), eGFR, uric acid, and lipid profiles, use of anti-platelet therapy, diuretics, statin, renin-angiotensin system inhibitor, and anti-hypoglycemic agent. Statistical significance was set as *P* < 0.05. All analyses were conducted using SAS (version 9.2; SAS Institute, Cary, NC, USA).

## Results

During 17,000.4 person-years of follow-up, 579 incident cases of composite renal endpoint developed (3.4 per 100 patients-year). There were 4987 (55.5%) and 4005 (44.5%) patients treated with empagliflozin and dapagliflozin, respectively. At baseline, the mean (SD) age, baseline BMI, and eGFR for the study cohort were 58.8 (11.3) years of age, 27.9 (4.7) kg/m^2^, and 93.3 (22.0) ml/min/1.73 m^2^, respectively. Overall, a mean (SD) BW loss of 1.2 (2.8) kg (− 1.65 $\pm$ 3.84%) was noted in the study patients following 3.04 $\pm$ 1.02 months of SGLT2i treatment (Table 1). The median following-up period of the study group was 24.3 [17.0, 29.6] months in the present study.

### Baseline characteristics of study population stratified by different baseline BMI or body weight loss following SGLT2i treatment

Additional file 1: Table SI summarizes the clinical characteristics of study population stratified by BMI. Compared to those with normal weight, patients with a higher baseline BMI were younger and had a shorter T2DM duration but a higher prevalence of hypertension and dyslipidemia. Obese population showed a lower HbA1c and a higher eGFR than those with normal weight. Obese patients were more likely to use statin and renin-angiotensin system inhibitor, but less likely to receive most hypoglycemic agents except metformin and glitazone.

Additional file 1: Table SII summarizes the clinical characteristics of patients stratified by the amount of BW loss. In general, patients with more BW loss were older; had a higher prevalence of female in gender, ischemic heart disease, congestive heart failure, stroke, and had a lower baseline eGFR. Moreover, a higher percentage of them received antiplatelet agent and diuretics, but a lower percentage of them received most hypoglycemic agents except insulin.

### Predictive baseline factors for a BW loss of ≥ 10.0% following SGLT2i treatment

The multivariate analysis indicated that the presence of congestive heart failure, use of diuretics, old age, female in gender, high-dose SGLT2i, and a higher aminotransferase level were independent factors associated with a BW loss of ≥ 10.0% associated with SGLT2i treatment (Additional file 2: Figure SI).

### Risk of composite renal outcomes study population stratified by different baseline BMI or body weight loss following SGLT2i treatment

**Fig. 2:**
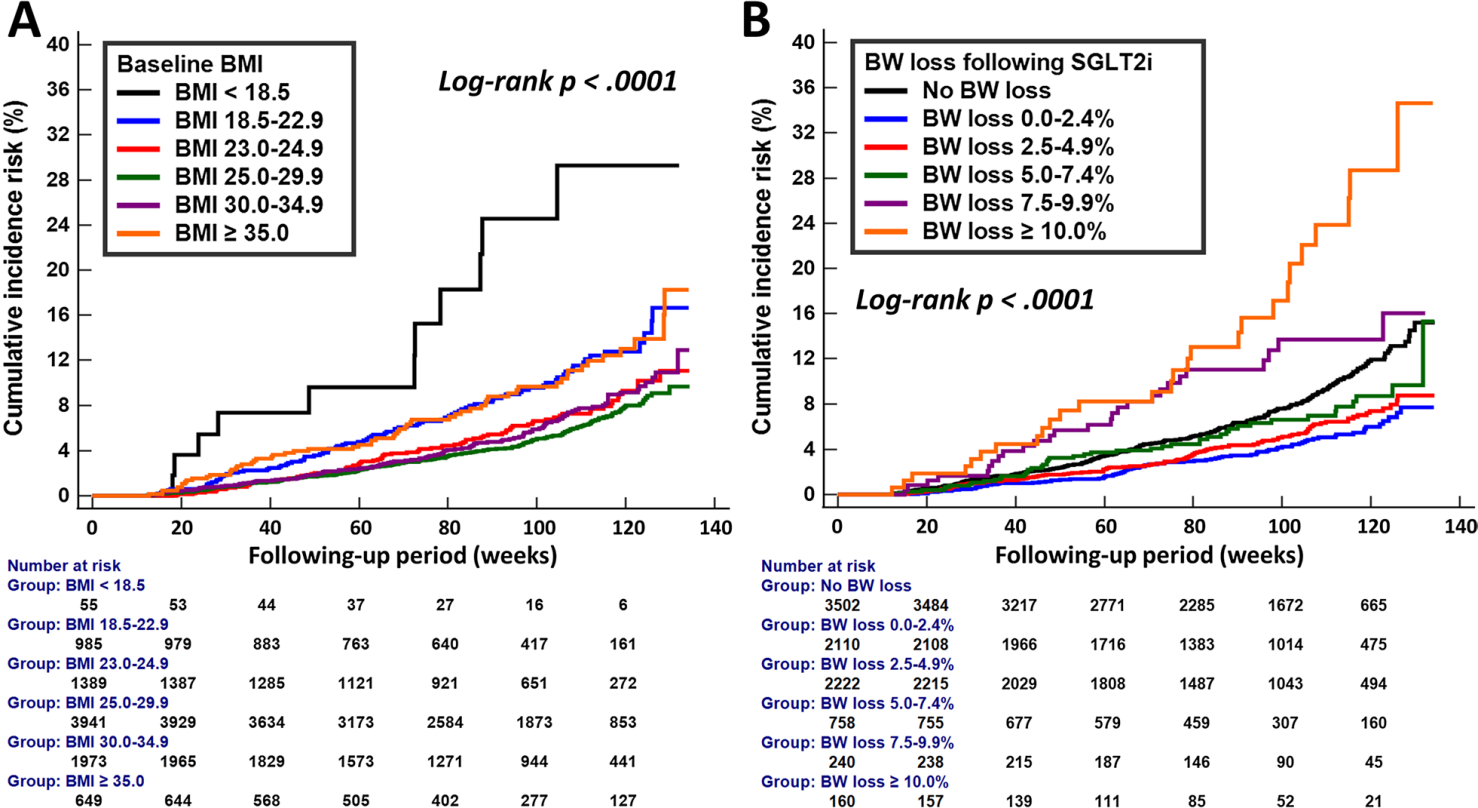
Cumulative incidence risk of composite renal outcome (sustained ≥ 40% decline in eGFR or the development of ESKD) for patients with T2DM in different categories of baseline BMI and initial BW loss associated with SGLT2i treatment. Patients with overweight (BMI: 23.0–24.9 kg/m^2^), pre-obese (BMI: 25.0–29.9 kg/m^2^), and obese I (BMI: 30.0–34.9 kg/m^2^) were associated with a substantially lower cumulative risk of composite renal outcome than those with a normal body weight at baseline (BMI: 18.5–22.9 kg/m^2^), whereas patients with underweight (BMI < 18.5 kg/m^2^) at baseline was associated with a higher cumulative risk of adverse renal event than those with a normal body weight at baseline (Log-rank *p* < 0.0001) (**A**). The cumulative risk of composite renal outcome appeared to be the lowest among patients with a modest BW loss of 0.0–2.4% and 2.5–4.9%, while the cumulative risk of composite renal outcome was the highest among patients with a pronounced BW loss of ≥ 10.0% (Log-rank *p* < 0.0001) (**B**). *BMI* body mass index, *BW* body weight, *eGFR* estimated glomerular filtration rate, *ESKD *end stage kidney disease, *SGLT2i* sodium glucose cotransporter 2 inhibitors, *T2DM* type 2 diabetes mellitus

The cumulative incidence curves of composite renal outcome of different categories of baseline BMI or BW change following SGLT2i treatment are shown in Fig. 2. Patients with overweight (BMI: 23.0–24.9 kg/m^2^), pre-obese (BMI: 25.0–29.9 kg/m^2^), and obese I (BMI: 30.0–34.9 kg/m^2^) were associated with a substantially lower cumulative risk of composite renal outcome than those with a normal body weight (BMI: 18.5–22.9 kg/m^2^) at baseline, whereas patients with underweight (BMI < 18.5 kg/m^2^) at baseline was associated with a higher cumulative risk of adverse renal event than those with a normal body weight at baseline (Log-rank *p* < 0.0001) (A). The cumulative risk of composite renal outcome appeared to be the lowest among patients with a modest BW loss of 0.0–2.4% and 2.5–4.9%, while the cumulative risk of composite renal outcome was the highest among patients with a pronounced BW loss of ≥ 10.0% (Log-rank *p* < 0.0001) (B).

**Fig. 3:**
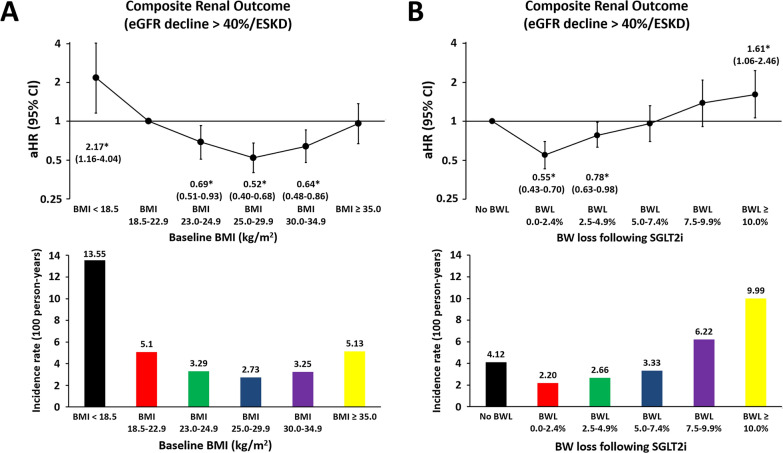
Risk of composite renal outcome for patients with T2DM in different categories of baseline BMI and initial BW loss associated with SGLT2i treatment. A U-shaped association between baseline BMI categories and development of composite renal outcome was observed with the lowest risk in the category of BMI of 25.0–29.9 kg/m^2^ and the highest risk in the category of underweight (BMI < 18.5 kg/m^2^) after multivariate adjustment (**A**). Compared with patients with no BW loss following SGLT2i treatment, a modest BW loss of 0.0–4.9% was associated with a significantly lower risk, whereas a pronounced BW loss ≥ 10.0% associated with a worse composite renal outcome after multivariate adjustment (**B**). Risk of study outcome was adjusted for age, gender, baseline comorbidities including history of ischemic heart disease, hypertension, stroke, heart failure, peripheral artery disease, gout, malignancy, and baseline BMI, urine ACR; HbA1c, eGFR, uric acid, and lipid profiles, use of anti-platelet therapy, diuretics, statin, renin-angiotensin system inhibitor, and anti-hypoglycemic agent. *ACR* albumin to creatinine ratio, *aHR *adjusted hazard ratio, *BMI* body mass index, *BW* body weight, *CI* confidence interval, *eGFR* estimated glomerular filtration rate, *ESKD* end stage kidney disease, *HbA1c* hemoglobin A1c, *SGLT2i* sodium glucose cotransporter 2 inhibitors, *T2DM* type 2 diabetes mellitus

We further examined the association between baseline BMI and BW change following SGLT2i treatment and composite renal outcome after multivariate adjustment of baseline characteristics (Fig. 3). Compared with patients with normal BMI (18.5–22.9 kg/m^2^), a U-shaped association between baseline BMI categories and development of composite renal outcome was observed with the lowest risk (adjusted hazard ratio (aHR): 0.52; [95% confidence intervals (CI): 0.40–0.68]) in the category of BMI = 25.0–29.9 and the highest risk (aHR: 2.17, [95% CI: 1.16–4.04]) in the category of underweight after multivariate adjustment (Fig. 3A). Compared with those with no BW loss associated with SGLT2i treatment, BW loss of 0.0–2.4% and 2.5–4.9% was associated with a significantly lower risk (aHR: 0.55; [95% CI: 0.43–0.70] for BW loss 0.0–2.4% and aHR: 0.78; [95% CI: 0.63–0.98] for BW loss 2.5–4.9%), whereas a BW loss ≥ 10.0% associated with a higher risk of composite renal outcome (aHR: 1.61; [95% CI: 1.06–2.46]) after multivariate adjustment (Fig. 3B).

### Sensitivity analysis

Among patients without BW loss associated with SGLT2i treatment (n = 3502), there were 1624 patients with BW gain during the study period, while there were 1878 patients with stable BW during the study period. Consistent with the main analysis, a U-shaped association between different BW loss and development of composite renal outcome was observed with the lowest risk in the category of BW loss 0.0–2.4% and the highest risk in the category of BW loss > 10.0% associated with SGLT2i treatment when compared with those with BW gain associated with SGLT2i treatment after multivariate adjustment (Additional file 3: Figure SII).

### Subgroup analysis

Subgroup analysis revealed that a modest decrease of 0.0–4.9% in BW associated with SGLT2i treatment was associated with a lower risk of composite renal outcome than no BW loss of 0.0–4.9% across all subgroups (*P* for interaction > 0.05; Additional file 4: Figure SIII). Also, a ≥ 10.0% decrease in BW associated with SGLT2i treatment was associated with a higher risk of composite renal outcome than BW loss < 10.0% associated with SGLT2i treatment across all subgroups (*P* for interaction > 0.05; Additional file 5: Figure SIV).

### Progression of albuminuria

**Fig. 4:**
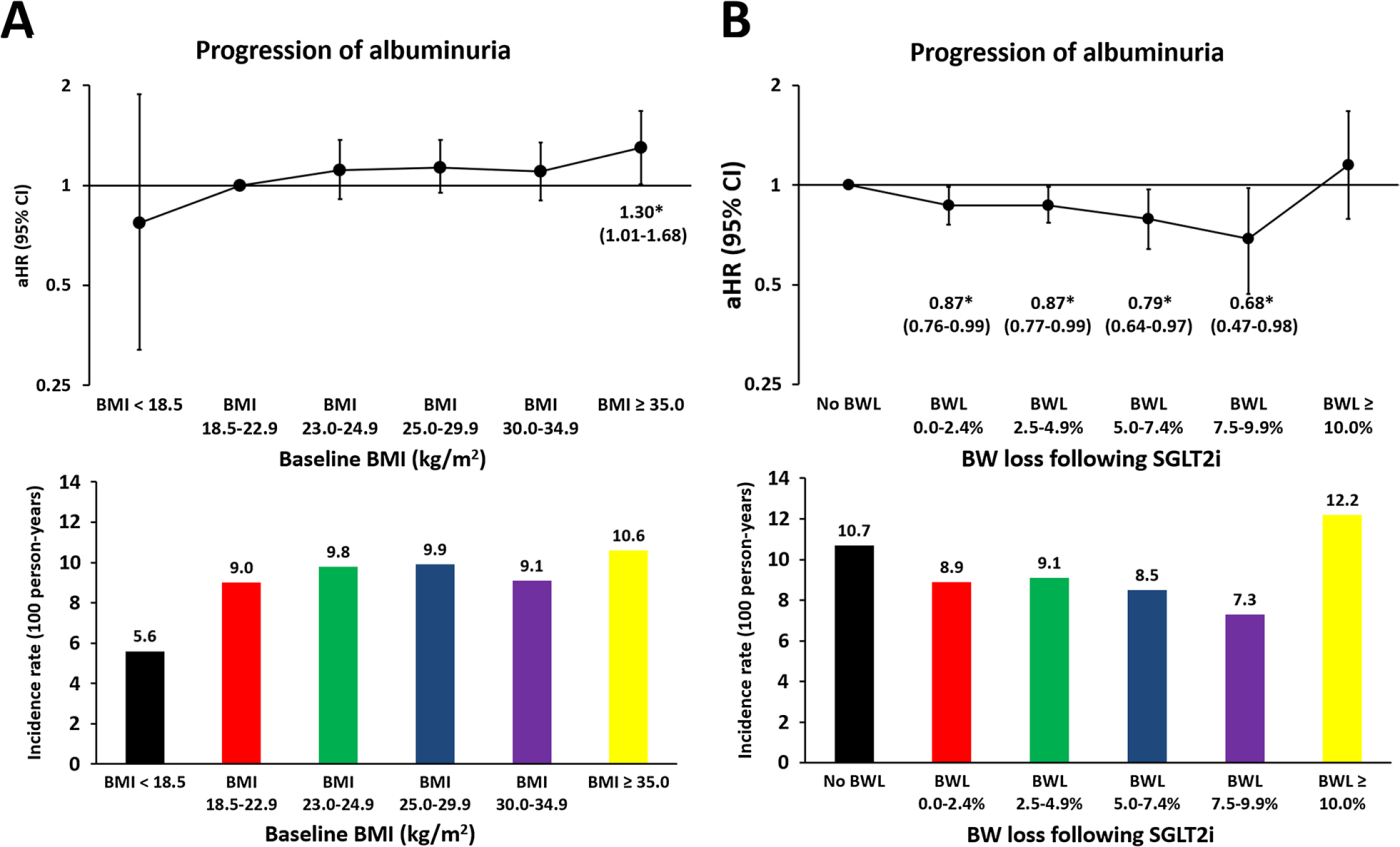
Risk of progression of albuminuria for patients with T2DM in different categories of baseline BMI and initial BW loss associated with SGLT2i treatment. Compared with patients with normal BMI (18.5–22.9 kg/m^2^), those with obesity II (BMI: ≥ 35.0 kg/m^2^) was associated with a higher risk of progression of albuminuria [new-onset microalbuminuria (urine albumin to creatinine ratio (ACR) > 30 mg/g) or macroalbuminuria (ACR > 300 mg/g)] after multivariate adjustment (**A**). Compared with those with no BW loss following SGLT2i treatment, BW loss of 0.0–2.4%, 2.5–4.9%, 5.0–7.5%, and 7.5–9.9% following SGLT2i treatment were all associated with a significantly lower risk of progression of albuminuria after multivariate adjustment (**B**). Adjusted risk factors of outcome as shown in Fig. 3. The abbreviations as in Fig. 1 to 3.

Compared with patients with normal BMI (18.5–22.9 kg/m^2^), those with obesity II (BMI: ≥ 35.0 kg/m^2^) was associated with a higher risk of progression of albuminuria (new-onset microalbuminuria (ACR > 30 mg/g) or macroalbuminuria (ACR > 300 mg/g)) after multivariate adjustment (Fig. 4A). Compared with those with no BW loss associated with SGLT2i treatment, BW loss of 0.0–2.4%, 2.5–4.9%, 5.0–7.5%, and 7.5–9.9% associated with SGLT2i treatment were all associated with a significantly lower risk of progression of albuminuria after multivariate adjustment (Fig. 4B).

## Discussion

An initial BW loss following treatment has been observed consistently across all SGLT2i outcome trials, but the implications of different BW loss following SGLT2i treatment on renal safety for patients with T2DM remained unclear. The main findings of this study showed a U-shaped association between baseline BMI categories and development of composite renal outcome with the lowest risk in the category of baseline BMI of 25.0–29.9 kg/m^2^ and the highest risk in the category of underweight (BMI < 18.5 kg/m^2^) after multivariate adjustment. Compared with patients with no BW loss associated with SGLT2i treatment, a modest BW loss of 0.0–4.9% was associated with a lower risk, whereas a pronounced BW loss ≥ 10.0% associated with SGLT2i treatment associated with a worse composite renal outcome after multivariate adjustment.

The associations between obesity and risk of adverse renal event have not been investigated in the population with T2DM extensively, and the results thus far are conflicting with each other. Some studies showed an positive and linear correlation between increased BMI and risk for ESKD [15, 17, 18]. Conversely, another large study reported an inverse association between BMI and CKD among 5829 Chinese patients with T2DM over a median follow-up duration of 4.6 years [20]. Recently, Kim et al. reported a U-shaped association between baseline BMI categories and development of ESKD with the lowest risk in the category of BMI 25.0–29.9 kg/m^2^ and the highest risk in the category of underweight (BMI < 18.5 kg/m^2^) among the 275,689 patients with T2DM diagnosed more than 5 years after multivariate adjustment [19].

Although extreme obesity is known to be associated with the risk of adverse kidney events, whether BW loss associated with a reduced risk of adverse renal outcome remained uncertain in patients with T2DM. Chung et al., studied 1187 diabetic patients aged 30–70 years at baseline (2003–2005), with follow-up surveys completed in 2008, 2009, and 2010. Among participants without CKD at baseline, those patients with a 5–10% BW loss had the lowest rate of eGFR decline [18]. Recently, the secondary analysis of the Look AHEAD (Action for Health in Diabetes) multicenter randomized clinical trial assessed whether an intensive lifestyle intervention (reduced caloric intake and increased physical activity) affects the development of incident nephropathy in people with T2DM. After an 8-year follow-up, the incidence of very high-risk CKD was lower in the intervention group with a modest BW loss of 5–10% at 1 year [26]. Although previous studies have reported the beneficial effects of BW loss in risk of adverse renal event on obese people with T2DM, a pronounced BW loss has been also reported as a risk factor for incident CKD in some studies [27, 28]. Our findings suggest that a modest BW loss of 0–5% (− 2.0 ± 1.0 kg) was associated with a favorable renal outcome, but a pronounced BW loss of ≥ 10% (− 10.1 ± 3.9 kg) may aggravate eGFR decline in patients with T2DM following SGLT2i treatment. These results suggest that the impact of BW change on kidney function may be determined by a balance between benefits and harms of BW loss to eGFR, which is dependent on the speed and amount of BW loss. Further prospective and randomized research clarifying our results is warranted.

Intentional BW loss may improve glycemic control and weight‐related comorbidities in the obese populations with T2DM, and in some cases, resulting in a long‐term remission of T2DM [29, 30]. The newer drug classes, including SGLT2i and glucagon‐like peptide receptor agonist (GLP‐1 RA), concomitantly improve glycemic control and resulted in BW loss, largely accounted for by body fat reduction [31]. In our present study, there were ~ 48% of patients with T2DM had experienced an initial BW loss of 0–5% (− 2.0 ± 1.0 kg) associated with SGLT2i treatment, which was generally consistent with the ~ 2–3 kg loss in BW reported in clinical trials [7–11]. Of note, our present study showed a total of 8.4%, 2.7%, and 1.8% of patients with T2DM had experienced an initial BW loss of 5.0–7.4% (− 4.5 ± 1.1 kg), 7.5–9.9% (− 6.1 ± 1.4 kg), and ≥ 10.0% (− 10.1 ± 3.9 kg) associated with around 12 weeks of SGLT2i treatment. Clinical trial data suggested that SGLT2i resulted in a mean BW loss of ~ 2–3 kg, but real‐world practice showed a significant heterogeneity in the magnitude of the BW loss following SGLT2i treatment [32]. SGLT2i treatment inhibit glucose reabsorption, promoting approximately 75 g of urinary glucose excretion with an associated caloric loss of around 300 Cal per day or 2100 Cal per week [33], which would be expected to be associated with a BW loss of ~ 0.5 kg loss per week and > 6 kg over 12 weeks of SGLT2i treatment (assuming no compensatory changes in energy balance). It has been speculated that the discrepancy between expected and observed BW loss following SGLT2i may arise because of compensatory increases in energy intake and changes in energy expenditure in order to minimize the energy imbalance [34]. Furthermore, SGLT2i caused an osmotic diuresis medicated by glycosuria and natriuresis, also resulting in an extra BW loss due to free water loss, with compensatory increases in fluid intake and vasopressin‐induced solute‐free water reabsorption to maintain body fluid balance [35]. Our present study showed that patients with a more pronounced BW loss were older; had a higher prevalence of female in gender, and several comorbidities including congestive heart failure or stroke (Additional file 1: Table SII). These fragile patients typically demonstrate early satiety and have poor food or fluid intake, which may result in a more pronouncing BW loss (either water or energy loss throughout the mechanism of SGLT2i) due to an impaired compensatory increase in energy and fluid intake. Hypovolemia substantially reduced the eGFR and may result in a worse renal outcome by reducing effective plasma volume and renal hypoperfusion. However, these issues regarding to the deteriorative kidney effect of a pronounced BW loss related to SGLT2i treatment required further elucidation in the future.

### Study limitations

This study has several limitations. First, this was a retrospective and observational study. The baseline characteristics of the study patients were different across different categories of baseline BMI or BW loss related to SGLT2i treatment. Although we have adjusted for several important parameters relevant to clinical outcomes in the multivariate adjusted Cox regression models, unidentified confounding variables that influence the effects of the clinical outcomes were still probably present (especially for the study subgroup with a pronounced BW loss of ≥ 10.0% associated with SGLT2i treatment). In the present study, whether the BW loss associated with the use of SGLT2i contributed from the loss of fat mass, lean body mass, or free water remained uncertain. Future prospective randomized studies are necessary to verify our findings. Second, the CGMH database is a closed medical system without external link in order to protect each patient’s privacy in CGMH. Therefore, it is impossible to obtain the medical activity of each patient outside the CGMH medical system in Taiwan, which may result in underestimation of medical activity of patients outside the CGMH system or loss of follow-up [36]. Third, we did not consider changes in the patients’ medical status or activity (e.g., discontinuation or add-on of co-medication or new diagnosis of comorbidities) during the follow-up period, which may also affect the outcome. Fourth, the follow-up period in the present study was relatively short to make us difficult confirm the impact of a variety of BW loss following SGLT2i treatment in long-term renal outcome. Future studies with longer following-up period are warranted to assess the benefit of SGLT2i in renal outcome among diabetic patients with a variety of BW loss following treatment. Fifth, we do not have data regarding the compliance of SGLT2i for the study population which may be even more pronounced in the subgroup without BW loss. Finally, the CGMH database is comprised mainly of Asian population, and whether the results in the present study can be extrapolated to non-Asian population remains unclear.

## Conclusions

A modest BW loss 0–5% associated with SGLT2i treatment was independently associated with a favorable renal outcome in diabetic patients. Caution should be taken for whom are underweight at baseline or have a pronounced BW loss $\ge$ 10.0% associated with SGLT2i treatment, which was associated with a worse renal outcome.

## Supplementary Information

**Additional file 1**: **Table SI**: Clinical characteristics of patients with type 2 diabetes mellitus (T2DM) treated with SGLT2i stratified by baseline BMI. **Table SII**: Clinical characteristics of patients with T2DM treated with SGLT2i stratified by changes in body weight (BW).

**Additional file 2**: **Figure SI**. Factors associated with ≥ 10.0% BW loss in patients treated with SGLT2i.

**Additional file 3**: **Figure SII**. Sensitivity analysis

**Additional file 4: Figure SIII. **Subgroup analysis of modest BW loss of 0.0-4.9% associated with SGLT2i treatment on risk of composite renal outcome in patients with T2DM.

**Additional file 5**: **Figure SIV**. Subgroup analysis of pronounced BW loss of ≥ 10% associated with SGLT2i treatment on risk of composite renal outcome in patients with T2DM.

## Abbreviations

ACEI: Angiotensin-converting enzyme inhibitor
ALT: Alanine aminotransferase
ARB: Angiotensin receptor blocker
AMI: Acute myocardial infarction
APT: Antiplatelet agent
BMI: Body mass index
BW: Body weight
CKD: Chronic kidney disease
DPP4i: Dipeptidyl peptidase-4 inhibitor
eGFR: Estimated glomerular filtration rate
ESKD: End stage kidney disease
HbA1c: Hemoglobin A1c
HDL: High-density lipoprotein
IHD: Ischemic heart disease
LDL: Low-density lipoprotein
PAD: Peripheral artery disease
SGLT2i: Sodium glucose cotransporter-2 inhibitor
SU: Sulfonylurea
T2DM: Type 2 diabetes mellitus

## Declarations

### Availability of data and materials

The datasets used in this study were only available in the Chang Gung Medical Data Center, Taiwan. The programs (codes) involved for this study are available from the corresponding author on reasonable request.
