# Supplementary material for: The risk of consequent nephropathy following initial weight loss in diabetic patients treated with sodium glucose cotransporter 2 inhibitors
Source: Cardiovasc Diabetol. 2021 Aug 16;20:167. doi: 10.1186/s12933-021-01361-z (PMC8369784; doi:10.1186/s12933-021-01361-z)
Supplement: Supplementary file 1 — Additional file 1: Table SI: Clinical characteristics of patients with type 2 diabetes mellitus (T2DM) treated with SGLT2i stratified by baseline BMI. Table SII: Clinical characteristics of patients with T2DM treated with SGLT2i stratified by changes in body weight (BW). [file 12933_2021_1361_MOESM1_ESM.docx]

**Supplemental Table I**

**Clinical characteristics of patients with type 2 diabetes mellitus (T2DM) treated with SGLT2i stratified by baseline BMI**

|  | **Underweight**  **BMI < 18.5**  **(n = 55)** | **Normal**  **BMI 18.5-22.9**  **(n = 985)** | **Overweight**  **BMI 23.0-24.9**  **(n = 1,389)** | **Pre-Obese**  **BMI 25.0-29.9**  **(n = 3,941)** | **Obese I**  **BMI 30.0-34.9**  **(n = 1,973)** | **Obese II**  **BMI** $\boldsymbol{\geq}$ **35.0**  **(n = 843)** |
| --- | --- | --- | --- | --- | --- | --- |
|  | **Clinical characteristics** | | | | | |
| **Diabetes duration (yr)** | 7.9±4.0 | 9.0 ±3.4 | 9.0 ±3.3 | 8.8 ±3.3 | 8.3 ±3.4 | 7.6 ±3.6 |
| **Age (yr)** | 62.3 ±11.2 | 61.9 ±10.6 | 61.6 ±10.5 | 59.6 ±10.7 | 55.8 ±11.4 | 52.2 ±12.1 |
| **Female** | 26 (47) | 500 (51) | 616 (44) | 1606 (41) | 816 (41) | 300 (46) |
| **Ischemic heart etiology** | 3 (5) | 70 (8) | 102 (7) | 300 (8) | 126 (6) | 32 (5) |
| **Hypertension** | 22 (40) | 502 (51) | 816 (59) | 2713 (69) | 1472 (75) | 507 (78) |
| **Dyslipidemia** | 26 (47) | 694 (70) | 1077 (78) | 3142 (80) | 1587 (80) | 500 (77) |
| **Cerebral vascular accidents** | 5 (9) | 38 (4) | 67 (5) | 159 (4) | 63 (3) | 29 (4) |
| **Congestive heart failure** | 5 (9) | 33 (3) | 47 (3) | 118 (4) | 60 (3) | 28 (4) |
| **Peripheral artery disease** | 1 (2) | 9 (1) | 16 (1) | 37 (1) | 6 (0) | 5 (1) |
| **Gout** | 3 (5) | 46 (5) | 104 (7) | 413 (10) | 272 (14) | 86 (13) |
| **Malignancy** | 6 (11) | 96 (10) | 134 (10) | 304 (8) | 145 (7) | 49 (8) |
|  | **Baseline body weight (BW) and BW change** | | | | | |
| **Baseline body weight (kg)** | 48.2 ±7.1 | 56.6 ±7.1 | 63.5 ±6.7 | 72.5 ±8.8 | 84.9 ±10.5 | 101.8 ±15.9 |
| **Baseline BMI (kg/m^2^)** | 17.5 ±0.9 | 21.6 ±1.1 | 24.1 ±0.6 | 27.3 ±1.4 | 31.9 ±1.4 | 38.5 ±4.2 |
| **Body weight loss (kg)** | -0.18 ±3.22 | -0.85 ±2.75 | -1.06 ±2.22 | -1.19 ±2.60 | -1.48 ±2.90 | -1.93 ±4.37 |
| **Body weight loss (%)** | -0.25 ±6.76 | -1.48 ±4.81 | -1.66 ±3.52 | -1.62 ±3.66 | -1.75 ±3.49 | -1.89 ±4.49 |
|  | **Baseline laboratory data** | | | | | |
| **HbA1c (%)** | 9.6 ±1.9 | 9.2 ±1.8 | 8.9 ±1.5 | 8.9 ±1.6 | 8.8 ±1.6 | 8.8 ±1.6 |
| **eGFR (ml/min/m^2^)** | 95.6 ±25.7 | 95.1 ±22.0 | 92.0 ±21.8 | 91.5 ±21.8 | 94.5 ±21.8 | 99.9 ±21.5 |
| **Triglycerides (mg/dL)** | 113.9 ±59.3 | 140.3 ±167.0 | 153.1 ±137.1 | 187.1 ±229.5 | 201.9 ±226.0 | 197.1 ±129.5 |
| **LDL (mg/dL)** | 91.4 ±32.9 | 93.2 ±30.5 | 92.1 ±29.9 | 91.7 ±29.9 | 93.1 ±28.9 | 95.6 ±28.8 |
| **HDL (mg/d)** | 49.9 ±18.7 | 48.1 ±13.6 | 45.1 ±11.2 | 43.3 ±10.6 | 42.5 ±10.5 | 42.1 ±9.9 |
| **Uric acid (mg/dL)** | 5.0 ±1.7 | 5.2 ±1.4 | 5.5 ±1.4 | 5.8 ±1.4 | 5.9 ±1.4 | 6.1 ±1.4 |
| **Urine ACR (mg/g)** | 487.7 ±1195.8 | 184.3 ±602.0 | 194.3 ±626.3 | 231.8 ±740.5 | 286.4 ±844.4 | 346.4 ±979.1 |
|  | **Baseline medications** | | | | | |
| **Anti-platelet agent** | 19 (35) | 263 (27) | 416 (30) | 1275 (32) | 636 (32) | 200 (31) |
| **Statin** | 27 (49) | 545 (55) | 871 (63) | 2574 (65) | 1257 (64) | 429 (66) |
| **ACEI or ARB** | 21 (38) | 419 (43) | 715 (51) | 2399 (61) | 1362 (69) | 455 (70) |
| **Use of diuretics** | 5 (9) | 71 (7) | 94 (7) | 325 (8) | 197 (10) | 95 (15) |
| **Anti-diabetic agent** |  |  |  |  |  |  |
| **SU** | 42 (76) | 705 (72) | 1005 (72) | 2690 (68) | 1341 (68) | 420 (65) |
| **Metformin** | 46 (84) | 880 (89) | 1263 (91) | 3610 (92) | 1826 (93) | 604 (93) |
| **Glinide** | 6 (11) | 39 (4) | 40 (3) | 138 (4) | 57 (3) | 17 (3) |
| **DPP4i** | 26 (47) | 543 (55) | 744 (54) | 2014 (51) | 941 (48) | 296 (46) |
| **Glitazone** | 11 (20) | 205 (21) | 331 (24) | 1049 (27) | 558 (28) | 186 (29) |
| **Acarbose** | 10 (18) | 229 (23) | 315 (23) | 864 (22) | 413 (21) | 124 (19) |
| **Insulin** | 15 (27) | 223 (23) | 258 (19) | 764 (19) | 356 (18) | 120 (18) |
| **GLP1 agonist** | 0 (0) | 2 (0) | 9 (1) | 27 (1) | 29 (1) | 17 (3) |

ACEI = angiotensin-converting enzyme inhibitor; ACR = albumin to creatinine ratio; ARB = angiotensin receptor blocker; BMI = body mass index; DPP4i = dipeptidyl peptidase-4 inhibitor; eGFR = estimated glomerular filtration rate; GLP1 = glucagon-like peptide 1; HBA1c = hemoglobin A1c; HDL = high-density lipoprotein; LDL = low-density lipoprotein; SGLT2i = sodium–glucose co-transporter-2 inhibitor; SU = sulfonylurea

Data are expressed as mean ± standard deviation or number (%).

**Supplemental Table II**

**Clinical characteristics of patients with T2DM treated with SGLT2i stratified by changes in body weight (BW)**

|  | **No BW loss**  **(n = 3,502)** | **BW loss**  **0.0-2.4%**  **(n = 2,110)** | **BW loss**  **2.5-4.9%**  **(n = 2,222)** | **BW loss**  **5.0-7.4%**  **(n = 758)** | **BW loss**  **7.5-9.9%**  **(n = 240)** | **BW loss**  $\boldsymbol{\geq}\boldsymbol{10}$**.0%**  **(n = 160)** |
| --- | --- | --- | --- | --- | --- | --- |
|  | **Clinical characteristics** | | | | | |
| **Diabetes duration (yr)** | 8.6 ±3.4 | 8.6 ±3.4 | 8.8 ±3.4 | 8.7 ±3.4 | 8.7 ±3.5 | 8.3 ±3.6 |
| **Age (yr)** | 58.5 ±11.4 | 57.6 ±11.0 | 59.4 ±11.0 | 59.6 ±11.2 | 61.0 ±11.3 | 62.6 ±13.5 |
| **Female** | 1462 (42) | 897 (43) | 974 (44) | 325 (43) | 121 (50) | 85 (53) |
| **Ischemic heart etiology** | 254 (7) | 138 (7) | 141 (6) | 65 (9) | 18 (8) | 17 (11) |
| **Hypertension** | 2354 (67) | 1450 (69) | 1447 (65) | 506 (67) | 173 (72) | 102 (64) |
| **Dyslipidemia** | 2696 (77) | 1685 (80) | 1768 (80) | 587 (77) | 184 (77) | 106 (66) |
| **Cerebral vascular accidents** | 154 (4) | 74 (4) | 76 (3) | 29 (4) | 13 (5) | 15 (9) |
| **Congestive heart failure** | 133 (4) | 46 (2) | 54 (2) | 22 (3) | 17 (7) | 19 (12) |
| **Peripheral artery disease** | 29 (1) | 16 (1) | 20 (1) | 1 (0) | 5 (2) | 3 (2) |
| **Gout** | 379 (11) | 210 (10) | 236 (11) | 62 (8) | 22 (9) | 15 (9) |
| **Malignancy** | 294 (8) | 172 (8) | 177 (8) | 64 (8) | 17 (7) | 10 (6) |
|  | **Baseline body weight (BW) and BW change** | | | | | |
| **Baseline body weight (kg)** | 72.8 ±14.9 | 76.3 ±15.7 | 74.1 ±14.0 | 73.9 ±15.6 | 72.8 ±15.5 | 72.5 ±16.0 |
| **Baseline BMI (kg/m^2^)** | 27.6 ±4.6 | 28.6 ±5.0 | 27.9 ±4.3 | 27.9 ±4.7 | 27.9 ±4.8 | 27.8 ±5.2 |
| **Body weight loss (kg)** | 1.09 ±2.15 | -1.21 ±0.43 | -2.69 ±0.75 | -4.46 ±1.08 | -6.08 ±1.42 | -10.11 ±3.86 |
| **Body weight loss (%)** | 1.53 ±3.15 | -1.60 ±0.41 | -3.63 ±0.73 | -6.03 ±0.61 | -8.36 ±0.78 | -13.88 ±3.68 |
|  | **Baseline laboratory data** | | | | | |
| **HbA1c (%)** | 9.1 ±1.8 | 8.8 ±1.6 | 8.7 ±1.4 | 8.7 ±1.5 | 9.0 ±1.7 | 9.0 ±1.9 |
| **eGFR (ml/min/m^2^)** | 92.4 ±22.7 | 94.7 ±21.3 | 94.1 ±21.1 | 92.5 ±22.4 | 91.5 ±22.1 | 88.7 ±24.2 |
| **Triglycerides (mg/dL)** | 188.7 ±219.6 | 185.1 ±222.0 | 171.7 ±189.4 | 166.5 ±159.6 | 152.3 ±99.2 | 157.8 ±108.9 |
| **LDL (mg/dL)** | 93.3 ±31.7 | 92.1 ±27.7 | 91.7 ±28.6 | 91.9 ±28.0 | 90.3 ±30.0 | 97.4 ±33.2 |
| **HDL (mg/dL)** | 43.5 ±11.3 | 43.5 ±10.9 | 44.5 ±11.1 | 44.8 ±11.3 | 45.1 ±11.5 | 43.3 ±11.9 |
| **Uric acid (mg/dL)** | 5.8 ±1.4 | 5.8 ±1.3 | 5.7 ±1.3 | 5.7 ±1.5 | 5.6 ±1.5 | 5.7 ±1.9 |
| **Urine ACR (mg/g)** | 263.1 ±775.9 | 241.0 ±784.0 | 209.7 ±675.3 | 219.6 ±807.3 | 231.0 ±635.6 | 390.9 ±1026.0 |
|  | **Baseline medications** | | | | | |
| **Anti-platelet agent** | 1083 (31) | 643 (30) | 686 (31) | 246 (32) | 88 (37) | 63 (39) |
| **Statin** | 2156 (62) | 1375 (65) | 1437 (65) | 486 (64) | 157 (65) | 92 (58) |
| **ACEI or ARB** | 2105 (60) | 1297 (61) | 1287 (58) | 432 (57) | 151 (63) | 99 (62) |
| **Use of diuretics** | 335 (10) | 159 (8) | 163 (7) | 68 (9) | 29 (12) | 33 (21) |
| **Anti-diabetic agent** |  |  |  |  |  |  |
| **SU** | 2387 (68) | 1450 (69) | 1553 (70) | 546 (72) | 171 (71) | 96 (60) |
| **Metformin** | 3163 (90) | 1967 (93) | 2045 (92) | 700 (92) | 221 (92) | 133 (83) |
| **Glinide** | 117 (3) | 78 (4) | 65 (3) | 22 (3) | 9 (4) | 6 (4) |
| **DPP4i** | 1711 (49) | 1109 (53) | 1144 (51) | 411 (54) | 112 (47) | 77 (48) |
| **Glitazone** | 858 (25) | 532 (25) | 628 (28) | 219 (29) | 70 (29) | 33 (21) |
| **Acarbose** | 738 (21) | 464 (22) | 502 (23) | 163 (22) | 52 (22) | 36 (23) |
| **Insulin** | 828 (24) | 371 (18) | 346 (16) | 111 (15) | 47 (20) | 33 (21) |
| **GLP1 agonist** | 33 (1) | 21 (1) | 18 (1) | 8 (1) | 3 (1) | 1 (1) |

ACEI = angiotensin-converting enzyme inhibitor; ACR = albumin to creatinine ratio; ARB = angiotensin receptor blocker; BMI = body mass index; DPP4i = dipeptidyl peptidase-4 inhibitor; eGFR = estimated glomerular filtration rate; GLP1 = glucagon-like peptide 1; HBA1c = hemoglobin A1c; HDL = high-density lipoprotein; LDL = low-density lipoprotein; SGLT2i = sodium–glucose co-transporter-2 inhibitor; SU = sulfonylurea

Data are expressed as mean ± standard deviation or number (%).

**Supplemental Material**

**Supplemental Figure Legends**

**Supplemental Figure I.** **Factors associated with *≥* 10.0% BW loss in patients treated with SGLT2i.**

The multivariate analysis indicated that the presence of congestive heart failure, use of diuretics, old age, female in gender, high-dose SGLT2i, and a higher aminotransferase level were independent factors associated with a BW loss of $\geq$ 10.0% following SGLT2i treatment**.**

ALT= aminotransferases; BMI = body mass index; BW = body weight; eGFR = estimated glomerular filtration rate; HbA1c = hemoglobin a1c; SGLT2i = sodium–glucose cotransporter 2 inhibitors; SU = sulfonyurea; T2DM = type 2 diabetes mellitus; TZD = thiazolidinedione

**Supplemental Figure II. Sensitivity analysis**

Among patients without BW loss associated with SGLT2i treatment (n = 3,502), there were 1,624 patients with BW gain during the study period, while there were 1,878 patients with stable BW during the study period. Consistent with the main analysis, a U-shaped association between different BW loss and development of composite renal outcome was observed with the lowest risk in the category of BW loss 0.0-2.4% and the highest risk in the category of BW loss > 10.0% associated with SGLT2i treatment when compared with those with BW gain associated with SGLT2i treatment after multivariate adjustment (*P* interaction all > 0.05).

aHR = adjusted hazard ratio; BW = body weight; CI = confidence interval

**Supplemental Figure III. Subgroup analysis of modest BW loss of 0.0-4.9% associated with SGLT2i treatment on risk of composite renal outcome in patients with T2DM.**

Subgroup analysis revealed that a 0.0-4.9% decrease in BW following SGLT2i treatment was associated with a lower risk of composite renal outcome than no BW loss of 0.0-4.9% across all subgroups.

ACEI = angiotensin-converting enzyme inhibitor; ARB = angiotensin receptor blocker; CHF = congestive heart failure; HTN = hypertension; IHD = ischemic heart disease

Other abbreviations as in **Supplemental Figure I.**

**Supplemental Figure IV. Subgroup analysis of pronounced BW loss of** $\boldsymbol{\geq}$ **10% associated with SGLT2i treatment on risk of composite renal outcome in patients with T2DM.**

A $\geq$ 10.0% decrease in BW following SGLT2i treatment was associated with a higher risk of composite renal outcome than BW loss < 10.0% following SGLT2i treatment across all subgroups (*P* interaction > 0.05).

The abbreviations as in **Supplemental Figure I to III.**
