# Supplementary figures and images for: The risk of consequent nephropathy following initial weight loss in diabetic patients treated with sodium glucose cotransporter 2 inhibitors
Source: Cardiovasc Diabetol. 2021 Aug 16;20:167. doi: 10.1186/s12933-021-01361-z (PMC8369784; doi:10.1186/s12933-021-01361-z)

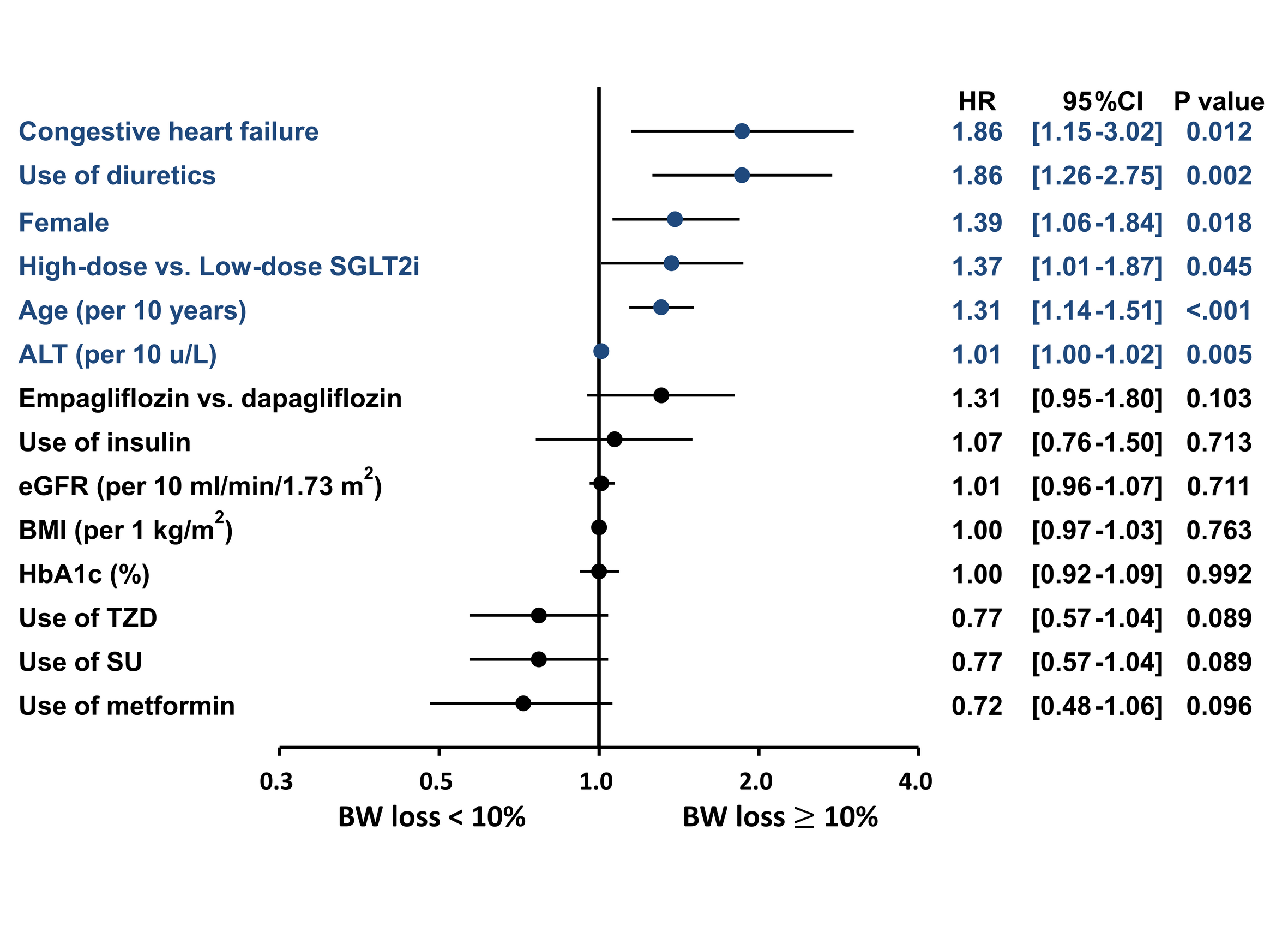

Supplement: Supplementary file 2 — Additional file 2: Figure SI. Factors associated with ≥ 10.0% BW loss in patients treated with SGLT2i. [file 12933_2021_1361_MOESM2_ESM.tif]

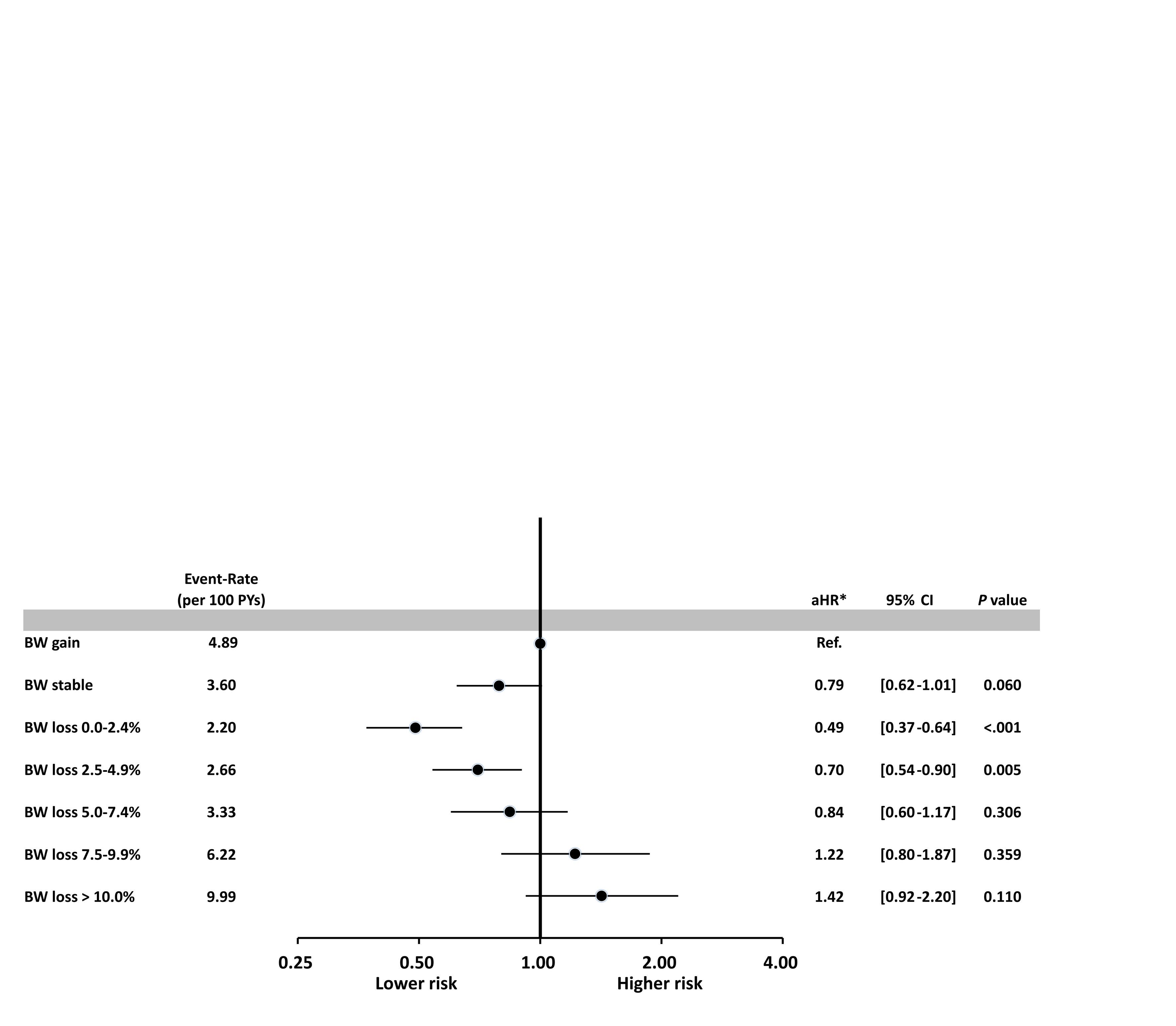

Supplement: Supplementary file 3 — Additional file 3: Figure SII. Sensitivity analysis [file 12933_2021_1361_MOESM3_ESM.tif]

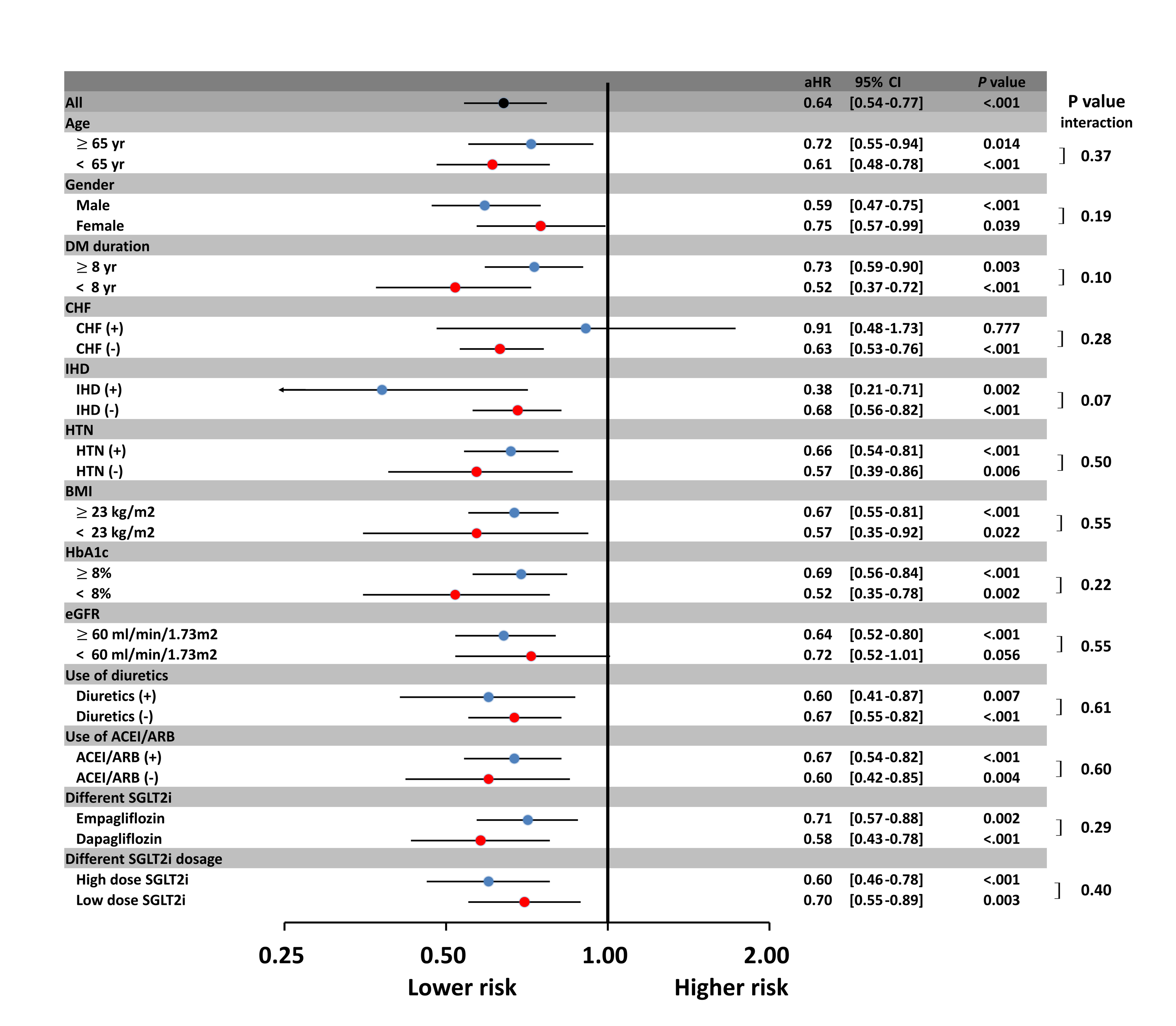

Supplement: Supplementary file 4 — Additional file 4: Figure SIII. Subgroup analysis of modest BW loss of 0.0-4.9% associated with SGLT2i treatment on risk of composite renal outcome in patients with T2DM. [file 12933_2021_1361_MOESM4_ESM.tif]

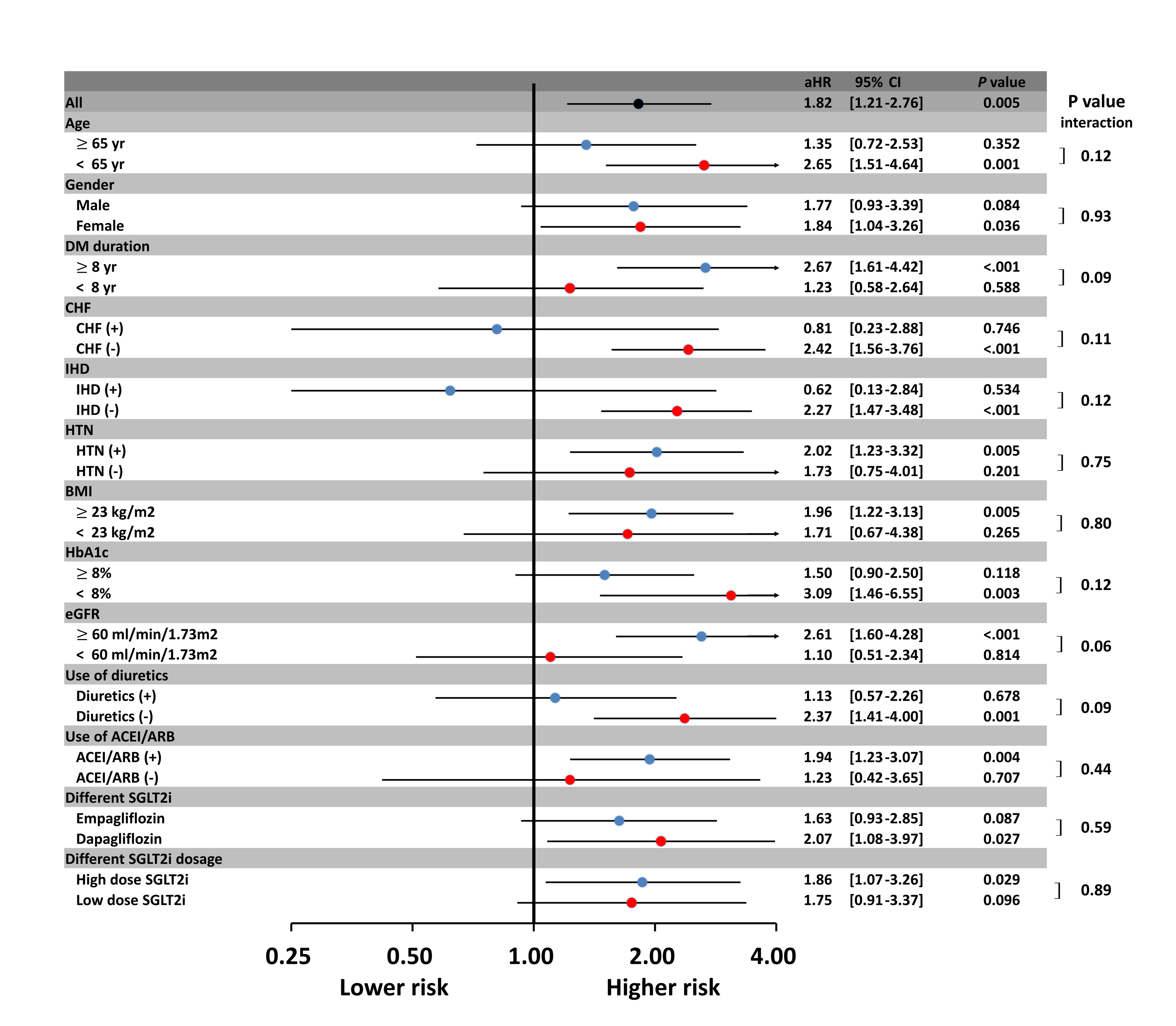

Supplement: Supplementary file 5 — Additional file 5: Figure SIV. Subgroup analysis of pronounced BW loss of ≥ 10% associated with SGLT2i treatment on risk of composite renal outcome in patients with T2DM. [file 12933_2021_1361_MOESM5_ESM.tif]
